# Quantum Dots for Wide Color Gamut Displays from Photoluminescence to Electroluminescence

**DOI:** 10.1186/s11671-017-1907-1

**Published:** 2017-02-27

**Authors:** Yongyin Kang, Zhicheng Song, Xiaofang Jiang, Xia Yin, Long Fang, Jing Gao, Yehua Su, Fei Zhao

**Affiliations:** 1 R&D Center, Nanjing Technology Corporation LTD., 500, Qiuyi Road, 4F, Building No.1, Binjiang District, Hangzhou, 310052 Zhejiang People’s Republic of China; 2 Multimedia R&D Center, Hisense, 399, Songling Road, Qingdao, 266100 People’s Republic of China

**Keywords:** Quantum dots (QDs), Quantum dot light-converting device (QLCD), Color filter (CF), Quantum dot light-emitting diode (QLED), Wide color gamut, Solution process

## Abstract

Monodisperse quantum dots (QDs) were prepared by low-temperature process. The remarkable narrow emission peak of the QDs helps the liquid crystal displays (LCD) and electroluminescence displays (QD light-emitting diode, QLED) to generate wide color gamut performance. The range of the color gamut for QD light-converting device (QLCD) is controlled by both the QDs and color filters (CFs) in LCD, and for QLED, the optimized color gamut is dominated by QD materials.

**Electronic supplementary material:**

The online version of this article (doi:10.1186/s11671-017-1907-1) contains supplementary material, which is available to authorized users.

## Background

Colloidal quantum dots (QDs) have been actively pursued for both fundamental research and industrial development due to their solution processibility and size-dependent optical properties associated with quantum confinement [1–4]. The most promising application of QDs is as emitters in biomedical labeling, solid-state lighting, and display [5–7]. For instance, in 2009, the US Department of Energy (DOE) highlighted a high-performance solid-state lighting device by NN-Labs LLC, in which QDs were embedded in polymer matrices and sandwiched in glass as a phosphor-converted LED (pc-LED) to produce high-quality warm-white lighting [8]. In the field of display, similar ideas show great potential to improve the visual performance, especially the wide color gamut of the devices [9–11].

Liquid crystal display (LCD) still dominates the market of medium- and large-sized screens for the advantages of low cost, long lifetime, relatively high ambient contrast ratio (ACR), such as monitors and TVs. As a necessary component in LCD panels, backlight is currently dominated by white LED backlight as a bright, stable, and economical solution. There are numerous ways to produce white light using LEDs, and the color gamut is determined by the contour of the emission peak from the phosphors. The conventional method uses a blue LED chip with YAG (yttrium-aluminum-garnet)-based phosphor directly packaged on its top, the color gamut is typically ~72% NTSC (National Television Standards Committee). Advanced phosphor-based technologies replace the YAG by green phosphors and the red phosphors, namely RG phosphor solution. In this relatively new solution, the color gamut can reach ~85% NTSC. In the past several years, QD-based backlight solutions have become available. The QD-based backlight solution takes the advantage of their extremely narrow full width at the half maximum (FWHM) of their photoluminescence [9, 11, 12]. Under typical conditions, the QD-based solution would readily reach ~100% NTSC. Furthermore, the NTSC in LCD display with QD-based backlight was strongly depended on the type of color filters (CF). A good “fitness” of CF and QD-based backlight could achieve 120–125% NTSC, while the “bad” CF only meet 90% NTSC, or even less [9, 13].

Meanwhile, QDs also are a promising new candidate for the emissive material in electroluminescence devices for display applications. Quantum dot light-emitting diodes (QLEDs) has advantages in color quality and stability especially when the fabrication of such devices was solution processing. This allows a considerable cost reduction and is therefore very attractive for industrial manufacturers. A. P. Alivisatos et. al. had taken the initial exploration for QD devices in 1994 [14]. And over the past few years, the performance QLED has been progressively improved, both in efficiency and lifetime. Unlike the full-color-enabling white QLED, the reported QLED here was in the form of monochromatic devices by the QDs with small FWHM. The small FWHM helped to improve color gamut significantly, and the range of color gamut was dominated by the synthesized QD materials.

Synthesis of QDs has gained tremendous attention in the past decades [15–18]. In principle, all emissive properties of QDs should be related to their excited states. By controlling their excited-state properties, QDs with near unity photoluminescence (PL) quantum yield (QY) and narrow FWHM (<20 nm) are synthesized through a low-temperature process [15, 18]. The photoluminescence QDs are applied to LCD displays named as quantum dot light-converting device (QLCD), and the electroluminescence QDs could be applied to QLED [2, 11]. Both the QLCD and QLED offer a high color gamut covering 100% rec. 2020 under optimal conditions. Since the QLED are fabricated with solution processes with low cost and high efficiency, it has attracted more attentions and would eventually bring a revolution in display fields [3].

## Methods

### Experimental

#### Chemicals/Materials

Cadmium oxide (99.99%), selenium (99.5%, 100 mesh), sulfur (99.98%, powder), trioctylphosphine oxide (TOPO, 90%), 1-octadecene (ODE), oleic acid (OA, 90%), stearic acid (99%), and myristic acid (99%) were purchased from Alfa-Aesar. Sodium diethyldithiocarbamate (NaDDTC · 3H_2_O, 99%) was purchased from Aladdin. All chemicals were used directly without any further purification. Cd(DDTC)_2_ was prepared by NaDDTC · 3H_2_O and CdAc_2_ · 2H_2_O. Cadmium diacetate dihydrate (CdAc_2_ · 2H_2_O) was purchased from Alfa-Aesar.

#### Synthesis of Nanocrystals

**Fig. 1:**
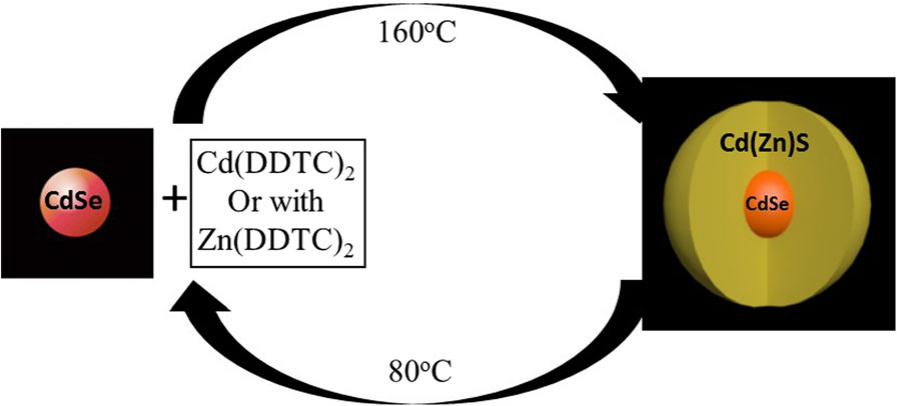
Schematic illustration of the synthetic process

The CdS/ZnS and CdSe/CdZnS or CdSe/CdS core/shell QDs were applied in this study. The related semiconductors are known to be in either zinc-blende (ZB) or wurtzite structures with small energy difference. This reminded us to develop low-temperature synthesis for highly crystalline nanocrystals. Figure 1 schematically illustrates the synthetic process of CdSe/CdZnS or CdSe/CdS. The core synthesis was critical to obtain the final narrow emission peak QDs. In a typical core synthesis, 20 mL of ODE, 0.64 g of CdO (5 mmol), and 2.5 g of myristic acid (11 mmol) were loaded into a 250 mL three-neck flask with stirring. The mixture was heated to 280 °C to obtain a colorless solution after bubbling of argon for 10 min. Five milliliter of the Se-suspension made by dissolving 1 mmol of Se in 30 mL of ODE was injected quickly into the above solution when the temperature was reduced to 250 °C. The temperature of the system was brought to 230 °C and then increase back to 250 °C for reaction. The continuous growth of the cores leads to a uniform optical properties. In the next step, more Se-suspension was dropping into the reaction vial with the temperature set at 250 °C. Prior to the injection of Se, 3 mmol oleic acid was dropwise added into the reaction flask at 20 mL/h and consequently, the reaction solution was allowed to react for 10 min under the given conditions. The same options were repeated until a targeted size of CdSe core was obtained. Needle-tip aliquots were taken and dissolved in toluene for UV–Vis and PL measurements to monitor the reaction. When the desired core was reached, the reaction was stopped by allowing the reaction mixture to cool down in air. An extraction procedure was used to purify the nanocrystals from side products and unreacted precursors.

In a typical shell synthesis, the purified core nanocrystals in ODE and about 4 mmol of cadmium diethyldithiocarbamate were loaded into 25 mL of ODE. After stirring and argon bubbling for 10 min, the mixture was heated to 160 °C and 7.5 mL of oleic acid was dropwise added. The reaction time after each dose was ∼10 min. Needle-tip aliquots were taken and dissolved in toluene for UV–Vis and PL measurements to monitor the reaction. When the desired shell thickness was reached, the reaction was stopped by allowing the reaction mixture to cool down in air.

#### Measurements and Characterization

UV–Vis spectra were taken on an Analytik Jena S600 UV–visible spectrophotometer. PL spectra were recorded by an Edinburgh Instruments FLS920 spectrometer. XRD measurements were carried out on a Rigaku Ultimate-IV X-ray diffractometer operated at 40 kV/30 mA with CuKα line (*λ* = 1.5418 Å). The size was achieved from the TEM (transmission electron microscope) images were taken on a Hitachi 7700 transmission electron microscope at 100 kV, and the nanocrystals were deposited onto an ultrathin carbon film on a copper grid.

#### Color Gamut Evaluation of the QDs in LCD Display and QLED

Serious CFs were selected from the known LCD companies. The color gamut was achieved from different groups of red-green-blue (RGB) QDs and CFs. CIE 1931 system was selected in the simulation process.

## Results and Discussion

### Quantum Dot with Different Emission Colors

The size of the core can be well controlled by fine tuning the addition of the precursors, and one can achieve QDs with different emission wavelengths by selecting the corresponding core and modifying the chemical composition of the shell. Table 1 summarizes the structural and photoluminescence properties of the QDs.

**Table 1:** Properties for QDs with different emission colors

System	Size (nm)	Optical parameters		
		PL (nm)	FWHM (nm)	QY (%)
CdSe/CdS	8–14	570–650	18	>90
CdSe/CdZnS	6–12	500–550	18	>90
CdS/ZnS	6–8	440–460	15	>85

The above QDs were synthesized by controlling their excited-state properties [18]. Fundamental studies on relationship between surface structure of semiconductor nanocrystals and their excited-state properties had been studied and attract full interest recently [15–18]. Key steps for achieving such optical quality include two related processes. Firstly, each nanocrystal must be grown with perfect crystal structure. In this step, single-source precursor is selected due to its significant activities at relative low temperature. Secondly, the surface of the QDs should be controlled to remove detrimental traps. Appropriate ligands always play an important role both as capping ligands and the deeply coupling with the surface elements. Atomic motion on the surface of the nanocrystals under enhanced ligand dynamics initiated intraparticle ripening without activating interparticle ripening. Ligand dynamics—i.e., ligands rapidly switching between being bonded to and detached from a nanocrystal upon thermal agitation—on nanocrystals to simultaneously retain colloidal stability and allow appreciable growth.

A “cleaning process” for the cores before the shell growth reaction is important. Typically, the reaction mixture was loaded into a glass vial and kept at 50–60 °C and into the flask, a mixture of methanol, acetone, and chloroform was added. The nanocrystal precipitated and was collected by centrifugation. The precipitate was then dissolved in a small amount of toluene, and another mixture of methanol, acetone, and chloroform was added. The same purification procedure was repeated twice, resulted in a clean QD cores. A clean surface of QDs always eliminates the excess of ligands and unreacted monomers which will suppress the crystallization defect in the following shell growth.

The corresponding PL emission peak of the QDs is shown in Additional file 1: Figure S1, which indicates an extremely narrow FWHM comparable with that of a corresponding single dot. For the TEM images in Additional file 1: Figure S2, the QDs show a nearly monodisperse morphology. To further confirm the crystal structure and overall phase purity, the nanocrystals with different sizes were examined using XRD. As shown in Additional file 1: Figure S3, all samples are found to be consistent with the expected diffraction pattern of the zinc-blende structures. From the peak width of the strongest peak, (111), the domain size of the particles was estimated using the Sherrer equation, which matched well with the particle sizes determined by TEM. To further confirm the crystallization, HRTEM images of the red QDs are shown in Additional file 1: Figure S2d, which revealed the single crystalline nature of the nanocrystals.

**Fig. 2:**
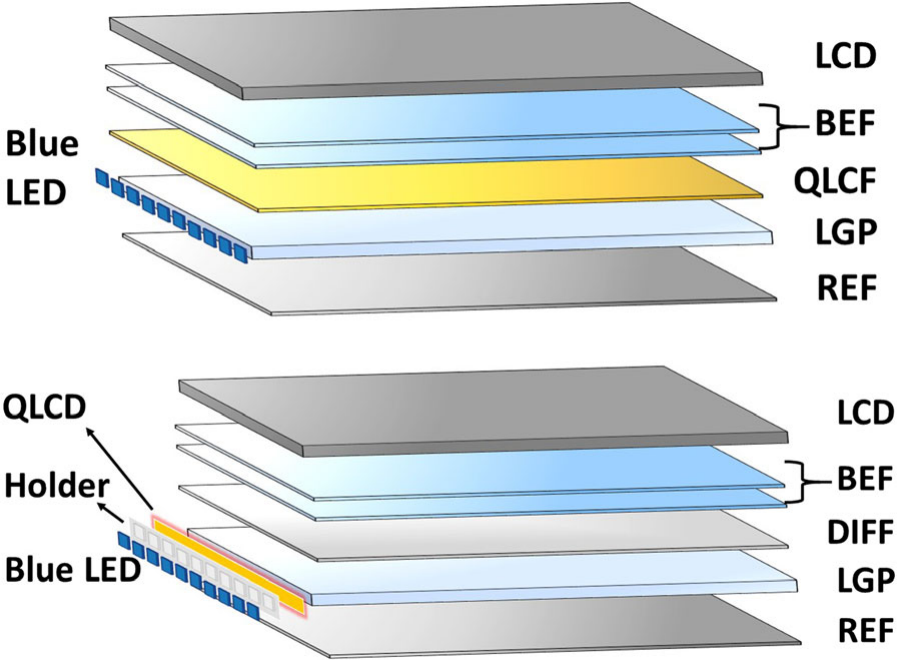
Schematic illustration of the structure for QLCD in LCD modules. *Top*: QD glass tube; *Bottom*: QD film

QLCD includes two commercial forms in the current LCD display market. One type is the QD in glass tube, where the green and red QDs are encapsulated into a sealed glass tube. Figure 2 (top image) indicates the working schematic diagram of QLCD in a typical LCD module. The QD glass tubes are excellent down-conversion emitters accompanied with the GaN-based blue LEDs. The glass could prevent the permanent of oxygen or moisture into QDs efficiently. Furthermore, the glass could survive from a high temperature even over 100 °C, which allows the QD glass tube to be close to the LED light sources. As a result, less QDs are needed in one glass tube that contributes to a low cost for the device. The moisture had been reported to be one of the reasons for the depredation of QDs through the surface interaction. This also needs a strong photo-reliability for QDs at such high temperature, i.e., the anti-blinking and anti-bleaching performance. Due to the restriction of the shape, the glass tube had to be fixed in the holders and could only be applied on TV backlight as on-edge method.

**Fig. 3:**
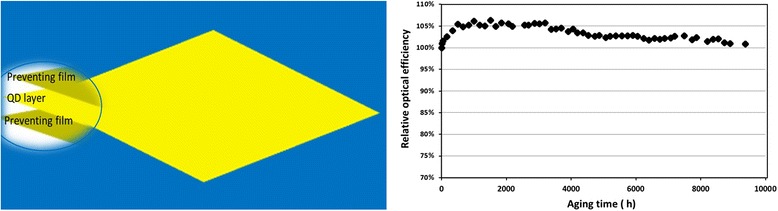
**a** A schematic image of the QLCF. **b** Time-dependent optical performance for QD films. Aging test condition: 70 °C with a blue light intensity of 0.15 W/cm^2^

Another type of QLCD is quantum dot light-converting film (QLCF) and the assembly structure in LCD module is illustrated in Fig. 2 (bottom image). The QLCF can be easily inserted in to the LCD backlight modules and fits for either on-edge LED or on-surface LED structures. The typical structure of QD film includes two preventing films and one QD layers (see Fig. 3a). If the conventional CdSe/CdS core/shell QDs were directly exposed in air without polymer or other protection, they went through photo-bleaching rapidly. The preventing films were introduced to suppress the penetration speed of moisture and oxygen into the emission materials. However, the preventing films would not work well at related higher temperature such as 90 °C or more. In another word, the QD film could not be closer to the LED sources and a large area of films would be used compared with the glass tube, resulted in a higher cost. However, the lower temperature applied on the QDs increases the reliability of the QD films. The accelerated aging test data shown in Fig. 3b demonstrates an aging time over 9000 h, which corresponds to ~100,000 h in a typical device’s shelf life. The outstanding reliability of the QD film is also contributed by the anti-bleaching properties of the as-prepared QDs with uniform shells enabled their excellent non-blinking behavior in air.

Both the QD glass tube and QD films provide wide color gamut when applied in a LCD display and the range of color gamut will be strongly depend on the QD materials, especially the QDs with narrow FWHM. A series of QDs with different emission positions were incorporated to QLCD, and the optimized peaks were selected to match the color filter settings of LCD panels. It is known that color filter in LCD panels to some extent will suppress the color purities of QDs because of the absorption overlapping of RGB regions and leakage of impurity light. Thick color filters can avoid overlapping of absorption spectra by eliminating light leakages. In contrast, power consumption increases as panel transmittance is lowered.

### The Color Gamut in LCD Displays with QLCD Will Be Dominated by both the QDs and the CFs

**Fig. 4:**
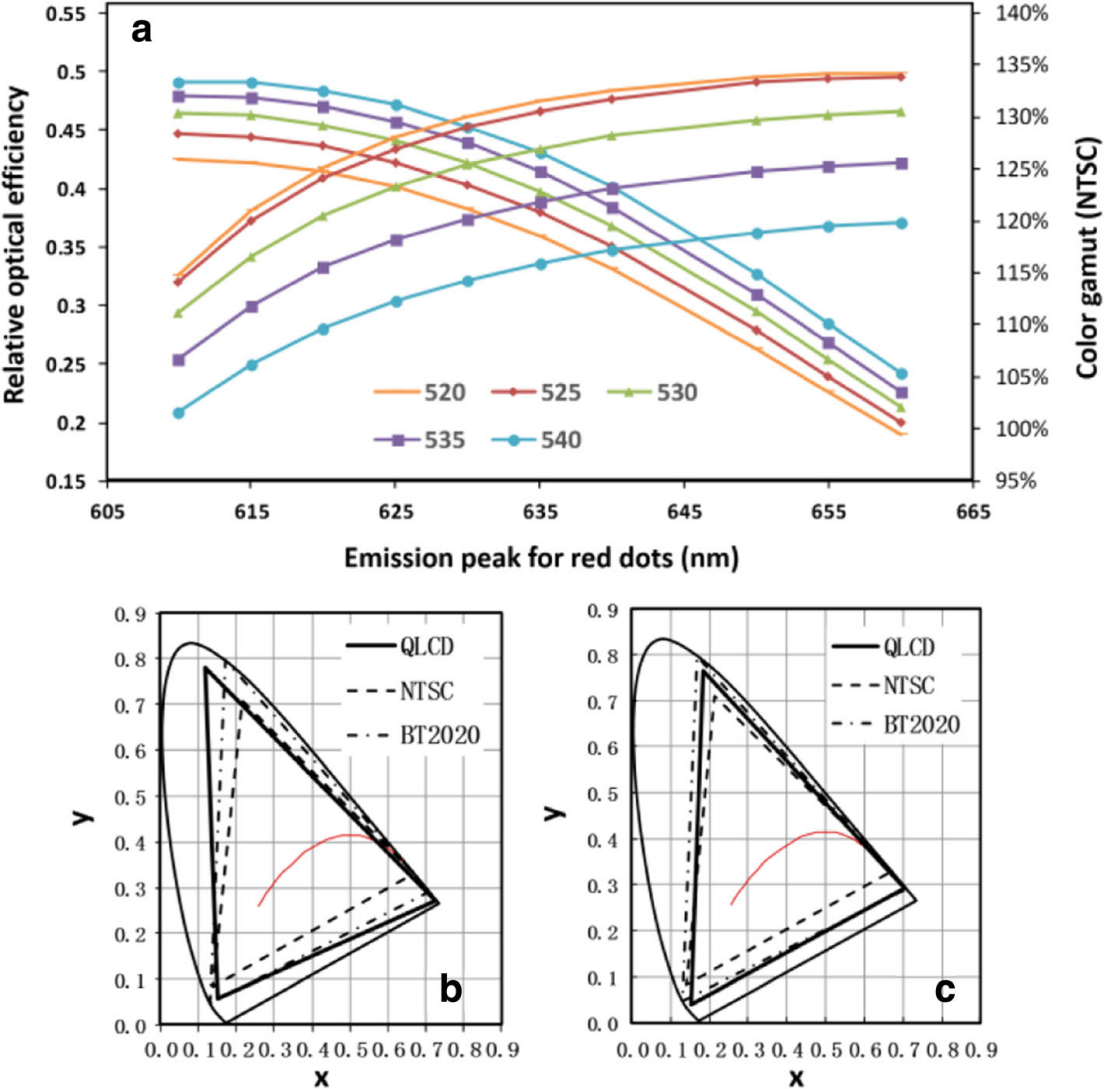
Wide color gamut from QLCD. **a** The emission peak dependence of the optical efficiency and the color gamut. **b** Maximum color gamut value in the experimental system. **c** The optimal color gamut in the experimental system. The standard of NTSC or rec. 2020 locate is for comparison

In this report, we selected several different commercial color filters to check the influence from the cross interactions between QDs and CFs. To avoid the mismatch, these CFs had similar absorption peak and just had different transmittance and absorption overlap for RGB range. In order to find the optimized QDs which had best match with CFs, the CF with better transmittance and relative lower absorption overlapping was selected for simulation. Figure 4 shows the trend of optical efficiency and color gamut value along with tuning PL peak positions.

In Fig. 4a, green-emitting QDs with FWHM of 18 nm were selected and we gained the target values by tuning the emission peak of the red-emitting QD with FWHM of 18 nm. From the results in Fig. 4, one can conclude that the emission peak position affects both color gamut and optical efficiency. By considering these two parameters, we found the proper peak positions for green-emitting and red-emitting QDs. Figure 4b shows the maximum color gamut value which could be achieved for the QLCD solutions using the QDs listed in Table 1, namely green emitting at 520 nm and red emitting at 635 nm. The corresponding color gamut was 134.5% NTSC (or 99.5% rec. 2020). However, the optical efficiency is very low. As an optimized choice, red emitting at 635 nm and green emitting at 530 nm provided a color gamut of 124.8% NTSC (or 92.3% rec. 2020) with a reasonable efficiency. In the manufacturing stage, the latter combination of green-emitting and red-emitting QDs would be suggested.

**Fig. 5:**
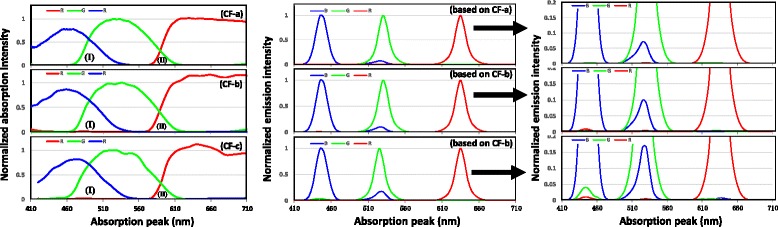
*Left*: absorption spectra for different CFs. (*I*) and (*II*) shows the overlap of GB and RG. *Middle*: emission spectra of RGB QDs after the absorption of the CFs. *Right*: the partial magnification of the RGB QD emissions in middle images

Upon the results above, we could check how the CF affect the color based on the same QDs. Figure 5 (left image) had provided the absorption spectra of three kinds of CFs, and Table 2 concluded the color gamut when the optimized QDs was applied. One can easily tell the significant gap from different groups of CFs. CF-a gives the highest color gamut, and CF-c offers a bad color gamut.

**Table 2:** Color gamut achieved from different CFs with optimized QDs

CFs	Color gamut with different QD groups			
	Red 635 nm	Green 520 nm	Red 635 nm	Green 530 nm
	NTSC	Rec. 2020	NTSC	Rec. 2020
CF-a (%)	134.5	99.5	124.8	92.3
CF-b (%)	125.5	91.4	115.2	83.9
CF-c (%)	110.0	80.1	101.0	73.6

Two factors were founded to affect the color gamut. One is FWHM of the QDs, the other one is the leakage of impurity light from the required color because of the cross absorption from green with blue (GB) and red with green (RG).This manuscript had provided the narrow FWHM QDs compared with the reported results. The emission spectra of the RGB QDs generated from the QLCD-based backlight will change as dedicated in the middle images in Fig. 5. The large overlap of the absorption (such as in (I) area in Fig. 5 left) will induce a leakage of green light to blue light or blue light to green light. More details could be founded in Fig. 5 right. For example, the green QD spectrum will have a small peak in blue and red range since the green CF allows the adjacent blue and red light pass through. Thus, the purity of the green light was smaller than the original light which would induce a smaller color gamut area. As along as the increase of overlap area from CF-a to CF-c, poor color gamut was acquired and indicated in Table 2.

As a commercial product, QLCD had attracted special attention in the market. For example, in 2015 SINOCES consumer electronics show, a Chinese TV manufacturer released a new product—K7100 new series ULED. It was the world’s first application of the quantum dot display technology in curved TV, which achieved a major breakthrough in the LCD TV picture quality with a 100% NTSC. Although the QD glass tube takes the advantage of the low cost than the QLCF, it could not fit for small display screens, such as mobile and pads, due to the large thickness. Furthermore, there is a sealed edge (ingress edge) for the glass tube which limit its application for super narrow border or no border TV designs. So, QD film turns more and more popular by the customers and the industries also put most of the efforts on making innovations for the low-cost QLCF.

### Quantum Dot Light-Emitting Diodes

**Fig. 6:**
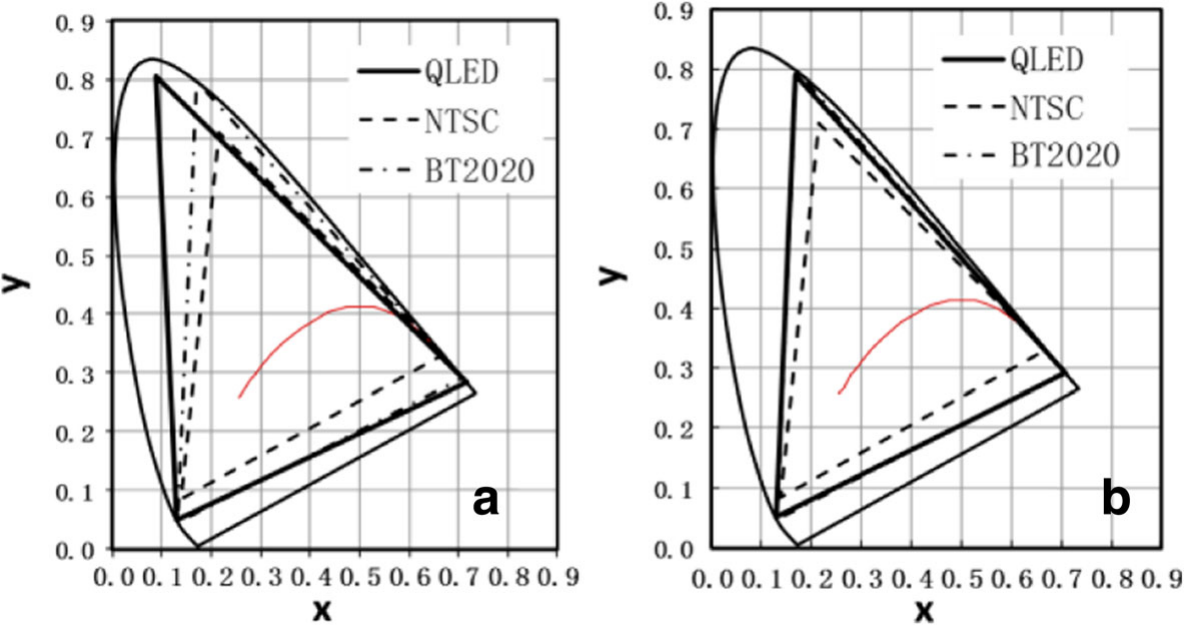
Wide color gamut from QLED. **a** Maximum color gamut value in the experimental system. **b** The optimal color gamut in the experimental system. The standard of NTSC or rec. 2020 locate is for comparison

**Fig. 7:**
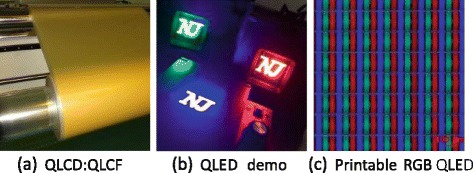
**a** QLCD with film types (QLCF) in the manufacturing lines. **b** QLED devices in operation. **c** RGB pixels in QLED by printing process

Quantum dot light-emitting diode (QLED) is becoming the most interesting alternative for the next generation of displays. Different from the LCD displays, the QLED would not need LED backlight and the relative CFs. The color expressed in QLED would only be dominated by the quality of QD materials. With the as-prepared QDs, we obtained QLEDs with solution processes. The external quantum efficiency of electroluminescence of the resulting devices was around 20, 18, and 14% for red, green, and blue QLEDs, respectively. Similar as the LCD display, one could obtain QLEDs a wide color gamut with electroluminescence peaks at 640 nm (red), 467 nm (blue), and 520 nm (green), namely 106.7% rec. 2020. The description of the color gamut is shown in Fig. 6a. Another set of QDs, i.e., with red emitting at 633 nm, blue emitting at 467 nm, and red emitting at 533 nm, presents a full-covered color gamut of rec. 2020 (Fig. 7b). Comparing these results with those in Fig. 3, one could readily see the negative impact of color filters due to the detrimental effects in LCD panels. In other words, QLED would offer perfect solutions of high optical quality for display.

Unlike the QLCD, the QLED has purer color quality without the restriction of C/F in LCD panels. One could acquire the desired color by tuning the emissions of the QDs, and the multi-color natural display system will be imagined.

### Solution Processing

QDs are solution processible materials, and the solution process will play a key role in the fabrication of QLCD and QLED devices. QLCF (see Fig. 7 left image) with flexible and thin properties is an important product type for QLCD which have been accepted by the main TV manufacturers. Currently, the roll-to-roll technologies have facilitated the manufacturing of the films, leading to low-cost, large-area, and high-efficiency QLCF. For QLED, our previous work had demonstrated the good performance of the solution-processed red LEDs with color-saturated emission, record efficiency, low-efficiency roll-off, sub-bandgap turn-on voltage, excellent reproducibility, and outstanding operational stability. Therefore, the overall performance was comparable to state-of-art OLEDs fabricated by vacuum deposition [6]. The presenting demos (seen in Fig. 7b) were prepared by following the procedures reported by Dai et. al [3]. A detailed process is provided in the supporting information. In the most recent work in our labs, ink jet printing was selected as an effective route for RGB QLED devices. All the synthetic method was similar as the typical procedure, except the QD layer, which was printing instead of spin coating. Figure 7c had demonstrated the details for a RGB QLED printing pixels, which make it possible for colorful QLED devices.

## Conclusions

High-quality QDs were synthesized by low-temperature process, and these QDs exhibit extremely narrow FWHM. The quantum efficiency for both PL and EL is high and thus supports the further development of QLCD and QLED devices. The remarkable photo-stability of the as-prepared QDs encourages the large-scale commercial manufacturing in display supply chain. Wide color gamut was achieved by applying the narrow QDs either with LCD or as QLED. CFs play an important role when acquiring the high color gamut in LCD display as well as the QD materials. The QD display is supposed to provide the best color solution for the current LCD market and the future QLED or OLED products.

## Additional file

Additional file 1: Figure S1. Photoluminescence of the as prepared Blue, Green and red nanoparticles. **Figure S2** TEM images of the QDs (a) blue QDs (b) Green QDs and (c) Red QDs and the HRTEM images of the red dots (d). **Figure S3** XRD patterns for the as-prepared red (a) green (b) and blue (c) QDs. the particle size of was estimated using the Sherrer equation and we got 6.5 nm for blue dots, 8.3 nm for green dots and 11.1 nm for red dots, which matched well with the TEM images. The peak shift also indicates the components for the shell structure. (DOCX 1105 kb)

## Abbreviations

CFs: Color filters
LCD: Liquid crystal display
QDs: Quantum dots
QLCF: Quantum dot light-converting film
QLED: Quantum dot light-emitting diode
TEM: Transmission electron microscope
