# Supplementary material for: Quantum Dots for Wide Color Gamut Displays from Photoluminescence to Electroluminescence
Source: Nanoscale Res Lett. 2017 Feb 27;12:154. doi: 10.1186/s11671-017-1907-1 (PMC5328897; doi:10.1186/s11671-017-1907-1)
Supplement: Additional file 1: Figure S1. — Photoluminescence of the as prepared Blue, Green and red nanoparticles. Figure S2 TEM images of the QDs (a) blue QDs (b) Green QDs and (c) Red QDs and the HRTEM images of the red dots (d). Figure S3 XRD patterns for the as-prepared red (a) green (b) and blue (c) QDs. the particle size of was estimated using the Sherrer equation and we got 6.5 nm for blue dots, 8.3 nm for green dots and 11.1 nm for red dots, which matched well with the TEM images. The peak shift also indicates the components for the shell structure. (DOCX 1105 kb) [file 11671_2017_1907_MOESM1_ESM.docx]

Supporting Information of

Quantum Dots for Wide Color Gamut Displays

From Photoluminescence to Electroluminescence

Yongyin Kang^1^, Zhicheng Song^2^*, Xiaofang Jiang^1^, Xia Yin^1^, Long Fang^1^, Jing Gao^1^,Yehua Su^1^ and Fei Zhao^1^*


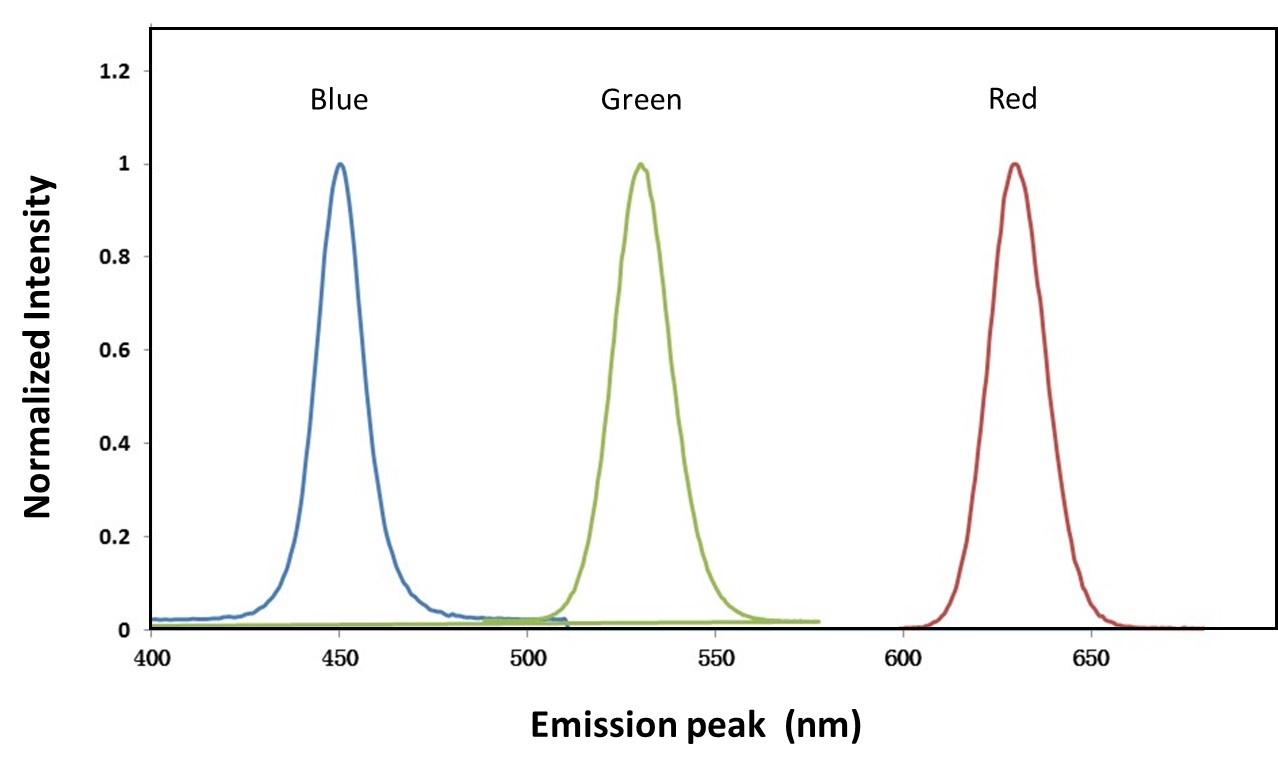


Fig. S1 Photoluminescence of the as prepared Blue, Green and red nanoparticles

**
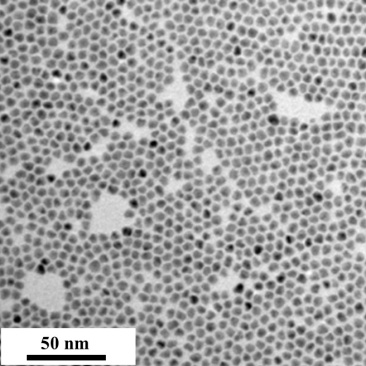
****
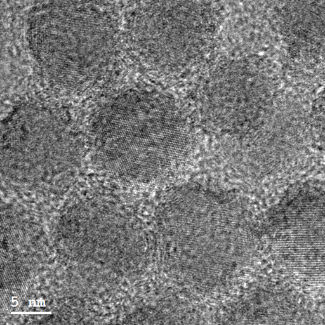
**

**(a) (b) (c) (d)**

Fig. S2 TEM images of the QDs (a) blue QDs (b) Green QDs and (c) Red QDs and the HRTEM images of the red dots (d)

Fig. S3 XRD patterns for the as-prepared red (a), green (b) and blue (c) QDs. the particle size of was estimated using the Sherrer equation and we got 6.5nm for blue dots, 8.3 nm for green dots and 11.1 nm for red dots, which matched well with the TEM images. The peak shift also indicates the components for the shell structure.

4. **The preparation of the QLED demo** follows the steps published by Dai. et. al. In a typical device fabrication process, ITO-coated glass was selected as substrates. The PEDOT:PSS solutions were spin-coated onto substrates at 3,000 r.p.m and baked at 140 °C for 10 min. then the PEDOT:PSS-coated substrates were transferred into the glove box. Poly-TPD, PVK, quantum dots, PMMA and ZnO nanocrystals were deposited layer by layer by spin coating at 3,000 r.p.m. before the deposition of the next layer. The poly-TPD and PVK layers were baked at 110 °C for 20min and at 170 °C for 30min, respectively. At the next step, Ag electrodes were deposited using a thermal evaporation system through a shadow mask under a high vacuum. The devices were encapsulated in the glove-box by the cover glasses using UV cured resin.
